# Phenotypic heterogeneity in metabolic traits among single cells of a rare bacterial species in its natural environment quantified with a combination of flow cell sorting and NanoSIMS

**DOI:** 10.3389/fmicb.2015.00243

**Published:** 2015-04-16

**Authors:** Matthias Zimmermann, Stéphane Escrig, Thomas Hübschmann, Mathias K. Kirf, Andreas Brand, R. Fredrik Inglis, Niculina Musat, Susann Müller, Anders Meibom, Martin Ackermann, Frank Schreiber

**Affiliations:** ^1^Department of Environmental Systems Sciences, ETH Zurich – Swiss Federal Institute of TechnologyZurich, Switzerland; ^2^Molecular Microbial Ecology Group, Department of Environmental Microbiology, Eawag – Swiss Federal Institute of Aquatic Science and TechnologyZurich, Switzerland; ^3^Laboratory for Biological Geochemistry, School of Architecture, Civil and Environmental Engineering, École Polytechnique Fédérale de LausanneLausanne, Switzerland; ^4^Department of Environmental Microbiology, Helmholtz-Centre for Environmental Research, LeipzigGermany; ^5^Department of Surface Waters, Eawag – Swiss Federal Institute of Aquatic Science and Technology, KastanienbaumSwitzerland; ^6^Department of Isotope Biogeochemistry, Helmholtz-Centre for Environmental Research, LeipzigGermany; ^7^Center for Advanced Surface Analysis, Institute of Earth Sciences, University of Lausanne, LausanneSwitzerland

**Keywords:** FACS, dinitrogen fixation, Lago di Cadagno, green sulfur bacteria, phenotypic noise, phenotypic variability, diversity, single-cell analysis

## Abstract

Populations of genetically identical microorganisms residing in the same environment can display marked variability in their phenotypic traits; this phenomenon is termed phenotypic heterogeneity. The relevance of such heterogeneity in natural habitats is unknown, because phenotypic characterization of a sufficient number of single cells of the same species in complex microbial communities is technically difficult. We report a procedure that allows to measure phenotypic heterogeneity in bacterial populations from natural environments, and use it to analyze N_2_ and CO_2_ fixation of single cells of the green sulfur bacterium *Chlorobium phaeobacteroides* from the meromictic lake Lago di Cadagno. We incubated lake water with ^15^N_2_ and ^13^CO_2_ under *in situ* conditions with and without NH_4_^+^. Subsequently, we used flow cell sorting with auto-fluorescence gating based on a pure culture isolate to concentrate *C. phaeobacteroides* from its natural abundance of 0.2% to now 26.5% of total bacteria. *C. phaeobacteroides* cells were identified using catalyzed-reporter deposition fluorescence *in situ* hybridization (CARD-FISH) targeting the 16S rRNA in the sorted population with a species-specific probe. In a last step, we used nanometer-scale secondary ion mass spectrometry to measure the incorporation ^15^N and ^13^C stable isotopes in more than 252 cells. We found that *C. phaeobacteroides* fixes N_2_ in the absence of NH_4_^+^, but not in the presence of NH_4_^+^ as has previously been suggested. N_2_ and CO_2_ fixation were heterogeneous among cells and positively correlated indicating that N_2_ and CO_2_ fixation activity interact and positively facilitate each other in individual cells. However, because CARD-FISH identification cannot detect genetic variability among cells of the same species, we cannot exclude genetic variability as a source for phenotypic heterogeneity in this natural population. Our study demonstrates the technical feasibility of measuring phenotypic heterogeneity in a rare bacterial species in its natural habitat, thus opening the door to study the occurrence and relevance of phenotypic heterogeneity in nature.

## Introduction

Research in the recent decade revealed that microbial cells of an isogenic population can show substantial variability in specific phenotypic traits even if they share the same environment (Raj and van Oudenaarden, 2008): this phenomenon is termed phenotypic heterogeneity. The inherent stochasticity of gene expression or cell-to cell variability in cellular components that globally affect gene expression [e.g., cell cycle proteins, number of ribosomes and polymerases, and ATP and NAD(P)H concentrations] can explain the emergence of variable phenotypes resulting in reproducible phenotype distributions in microbial populations with large numbers of individuals (Elowitz et al., 2002; Davidson and Surette, 2008). Furthermore, stochasticity of gene expression can be influenced by genetic factors. For example, changes in promoter sequences or ribosomal binding sites can alter binding affinities of transcription factors. As a consequence, the degree of phenotypic heterogeneity in a particular trait can be modulated by natural selection and change in the course of evolution (Ozbudak et al., 2002). This has raised the fundamental question whether phenotypic heterogeneity is beneficial and can provide adaptive functions. Division of labor and bet-hedging are two functions that can be mediated by phenotypic heterogeneity as has been proposed based on theoretical and experimental work with pure cultures (Balaban et al., 2004; Kussell and Leibler, 2005; Acar et al., 2008; Ackermann et al., 2008, Beaumont et al., 2009; Ratcliff and Denison, 2010; Ackermann, 2013; Arnoldini et al., 2014).

Molecular mechanisms and biological functions related to phenotypic heterogeneity have been investigated for a wide range of microbial traits including behavior (Korobkova et al., 2004; Emonet and Cluzel, 2008), stress response (Balaban et al., 2004; Maamar et al., 2007; Veening et al., 2008; Levy et al., 2012; Wakamoto et al., 2013) and metabolism (Ozbudak et al., 2004; Kiviet et al., 2014; Kotte et al., 2014; New et al., 2014; Solopova et al., 2014). Direct observation of growth, morphologies, quantification of intracellular compounds, and gene expression as measured by reporter-gene fusions are the main approaches to study phenotypic heterogeneity. Thus, studies have commonly focused on laboratory-grown cultures of either model organisms (Young et al., 2012) or libraries of wild isolates of the same species (Ziv et al., 2013; Holland et al., 2014; New et al., 2014). In contrast, determining phenotypic heterogeneity directly in the environment is demanding, because the established approaches cannot be employed there. Therefore, other quantitative single-cell methods need to be integrated to investigate phenotypic heterogeneity in the environment.

Nanometer-scale secondary ion mass spectrometry (NanoSIMS) is a powerful tool to measure the isotopic composition of single cells (Lechene et al., 2006; Hoppe et al., 2013). This allows determining the rate at which single cells assimilate isotopically labeled substrates into their biomass. NanoSIMS has been used in combination with 16S rRNA-based identification by catalyzed-reporter deposition fluorescence *in situ* hybridization (CARD-FISH) to link identity and function of microorganisms in their natural environment (Musat et al., 2012). These studies reported high levels of heterogeneity in metabolic activities of microbial populations identified with species-specific rRNA-targeted FISH probes (Lechene et al., 2007; Behrens et al., 2008; Musat et al., 2008; Halm et al., 2009; Woebken et al., 2012, 2014; Berry et al., 2013). It is important to note that natural cell populations detected with a species-specific FISH probe likely contain genetic variability (Thompson et al., 2005; Kashtan et al., 2014). Therefore, we use the term ‘phenotypic heterogeneity’ here in a broader sense than defined above including genetic variabiltiy as a source for phenotypic differences between individual cells.

The disadvantage of NanoSIMS is the low sample throughput (5–10 images per day), the high measurement costs, and the limited number of available instruments. These disadvantages represent a major obstacle for using NanoSIMS to quantify and further investigate the causes and consequences of phenotypic heterogeneity in complex microbial populations. The limitation of NanoSIMS especially applies to bacteria in complex environmental samples, because many species are part of the rare biosphere in communities with high diversity (relative abundance <0.1%; Sogin et al., 2006; Pedros-Alio, 2012). Conventional NanoSIMS sample preparation using filtration of the total community onto a filter membrane will lead to relatively few, interspaced cells of rare bacteria. A typical NanoSIMS image covering 35 μm × 35 μm contains about 100 cells (each about 1 μm long). Consequently, a species with a relative abundance of 1% would be represented with about a single cell per image, which leads to undesirable long measuring times to assess phenotypic heterogeneity even for a single sample. Flow cytometry combined with flow cell sorting (also known as fluorescence-activated cell sorting – FACS) provides the possibility to concentrate subpopulations residing in complex communities (Müller and Nebe-von-Caron, 2010; Lomas et al., 2011; Koch et al., 2013). Flow cell sorting has been combined with radioactive or stable isotope incubations to measure metabolic activities of specific functional groups on the level of sorted sub-populations (Zubkov et al., 2001, 2003) or on the single-cell level for a limited number of individuals (Thompson et al., 2012).

In this study, we present a procedure that allows quantification of phenotypic heterogeneity in metabolic activities of rare bacteria. We studied phenotypic heterogeneity in N_2_ and CO_2_ fixation in the green sulfur bacterium *Chlorobium phaeobacteroides* residing in the chemocline of the meromictic lake Lago di Cadagno. We chose *C. phaeobacteroides* in Lago di Cadagno, because an earlier study indicated strong heterogeneity in N_2_ fixation in this population based on the analysis of a relatively low number of cells with NanoSIMS (Halm et al., 2009). In addition, bulk ^15^N_2_ fixation measurements and molecular data suggested that *C. phaeobacteroides* fixes N_2_ in the presence of NH_4_^+^, despite the high metabolic costs of N_2_ fixation as compared to NH_4_^+^ assimilation. In the present study we sought to investigate this phenomenon in more detail and to develop a general approach to study phenotypic heterogeneity of rare microbial populations in the environment. The approach involved incubation of samples from the Lago di Cadagno chemocline with ^15^N_2_ and ^13^CO_2_. Then we used flow cell sorting with auto-fluorescence gating based on a pure culture isolate to enrich *C. phaeobacteroides*. Sorted cells were transferred to a filter membrane for subsequent NanoSIMS measurements to assess phenotypic heterogeneity in metabolic activities.

## Materials and Methods

### Study Site

Lago di Cadagno is a permanently stratified (meromictic) lake located in the Swiss Alps (1923 m above sea level). The maximum depth of the lake is 21 m. It is infiltrated through gypsum-rich, dolomite rock transporting salts including sulfate to the bottom-water. This process establishes an anaerobic, sulfidic hypolimnion with high salinity, and an aerobic, low-salinity epilimnion separated by a permanent chemocline in 10–14 m depth. The chemocline is characterized by sharp gradients and a strong turbidity maximum (ca. 1 m vertical thickness) dominated by populations of purple and green sulfur bacteria, which grow by anoxygenic photosynthesis with sulfide. The exact depth of the turbidity maximum can fluctuate as internal waves built up depending on the wind conditions. *In situ* measurements and stable isotope incubations were conducted in August, 2013 (bulk) and September, 2013 (NanoSIMS).

### Physicochemical Measurements

Physical and chemical properties of the water column were examined using a previously described Profiling *In situ* Analyzer (PIA) equipped with an oxygen microoptode, a sulfide sensor, a CTD (conductivity, temperature, depth) probe, and a carousel syringe sampler (Kirf et al., 2013). Ammonium (NH_4_^+^) and sulfide were measured as previously described (Cline, 1969; Holmes et al., 1999).

### Stable Isotope Incubation at Lago di Cadagno

**FIGURE 1:**
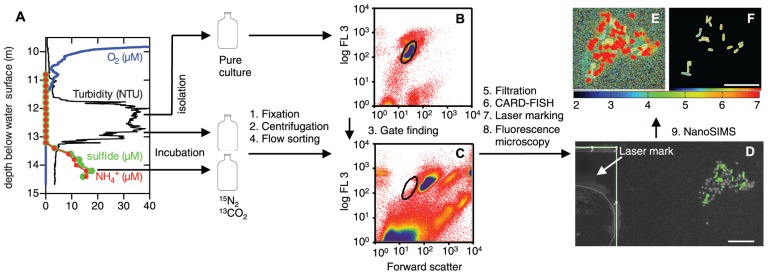
**Work flow from sampling to determination of phenotypic heterogeneity with nanometer-scale secondary ion mass spectrometry (NanoSIMS). (A)** *In situ* profiles of O_2_ (blue), turbidity (black), sulfide (green), and NH_4_^+^ (red) in Lago di Cadagno. The arrows indicate the sampling position for stable isotope incubations with and without NH_4_^+^ and isolation of a *Chlorobium phaeobacteroides* pure culture. After incubations, the samples were fixed with formaldehyde, washed by centrifugation, and subjected to flow cell sorting. **(B,C)** Flow cytometry plots of the *C. phaeobacteroides* pure culture **(B)** and the lake community **(C)** based on forward scatter (FSC) and auto-fluorescence (FL3; ex.: 488 nm; em.: 664 nm long-pass filter). The sorting gate (black circle) for the lake sample was determined from the pure culture plot. The color gradient from red to yellow to blue indicates increasing count density. Sorted cells that were placed on a membrane filter, hybridized with a CARD-FISH probe targeted against *C. phaeobacteroides* (green), and stained with Hoechst general DNA stain (gray) as shown in **(D)**. A mark etched with a laser into the membrane filters is shown in the white inset in **(D)**. The etched mark is the thick broken line underneath the thin gray line that digitally marks the drawing position of the laser. The mark is used to find the position of the imaged cells for NanoSIMS measurements. **(E,F)** ^15^N atom fractions [^12^C^15^N/(^12^C^14^N + ^12^C^15^N)]⋅10^-3^ according to the color scale calculated from nitrogen ion counts obtained with NanoSIMS for cells shown in **(D)**. **(E)** All data and **(F)** data for cells segmented on the basis of the CARD-FISH signal in **(D)**. Scale bars are 10 μm.

We investigated phenotypic heterogeneity in N_2_ and CO_2_ fixation in the green sulfur bacterium *C. phaeobacteroides* in the chemocline in the presence and absence of NH_4_^+^. Initial profiles showed that NH_4_^+^ repeatedly increased from concentrations below the detection limit (ca. 0.2 μM) to 5–15 μM right below the turbidity maximum (**Figure 1A**). We collected water samples at 12.9 m depth (within the turbidity maximum without NH_4_^+^) and at 14.2 m depth (below the turbidity maximum with 16.6 μM NH_4_^+^) in 570 ml serum flasks. The serum flasks were crimp sealed with blue rubber butyl stopper and made anoxic by five cycles of vacuum and rinsing with argon before use. The lake water was sampled directly into the anoxic serum flasks using oxygen-tight Viton-tubing and a peristaltic pump (IP 24, Ismatec). The serum flasks were kept cold and shaded from direct sunlight during sampling. The serum flasks were only filled to 4/5 of their volume with lake water. The incubation was started immediately after sampling in the on-site laboratory by filling the remaining volume with pre-prepared isotope-labeled pulse medium (preparation is described below). The final labeling percentage was 20% ^15^N_2_ and 2.8% ^13^CO_2_. The final NH_4_^+^ concentration was 13.5 μM in the treatment with NH_4_^+^ and below the detection limit (ca. 0.2 μM) in the treatment without NH_4_^+^. The samples were incubated under controlled temperature (6°C) and light (1.7 μmol Photons m^-2^ s^-1^ emitted by two fluorescent lamps, Radium BioSun Spectralux^®;^ NL-T8 36W/965/G13). The samples were incubated for 23 h containing a 9.75 h dark phase, which coincided with the natural night phase. NH_4_^+^ and sulfide concentrations were determined at the end of the incubation confirming that the concentration did not change significantly during the incubation.

^15^N_2_- and ^13^CO_2_-labeled pulse medium for the incubation experiment was prepared on the day before the incubation with modifications as described by Mohr et al. (2010). Water was sampled from 12.5 m (i.e., within the turbidity maximum without NH_4_^+^) depth into 2 L bottles using a peristaltic pump (Amicon LP-1 Peristaltic Pump with Masterflex 7015-21 Pump Head). The water was first pre-filtered (Durapore Membrane, HVLP, hydrophilic, 47 mm, 0.45 μm from Millipore) and then sterile-filtered (Durapore Membrane, HVLP, hydrophilic, 47 mm, 0.22 μm from Millipore). Next, the water was degassed by applying a vacuum during constant agitation for 1 h. The anoxic and gas-free medium was transferred anoxically into ethyl vinyl acetate infusion bags (Bioeaze, Sigma). Subsequently we added Na_2_S (150 μM final), ^15^N_2_ (17 mL L^-1^; Sigma, lot EB1169V) and ^13^C-NaHCO_3_ (182 μM final). The infusion bags were kept dark on a shaker overnight in a water bath to equilibrate the ^15^N_2_ within the liquid and to remove residual O_2_ by reaction with sulfide. Sulfide concentrations were measured shortly before starting the incubations and the sulfide lost by reaction with O_2_ was re-added.

The incubations were stopped by fixing 150 mL lake water in methanol-free 1% paraformaldehyde (Electron Microscopy Sciences) amended with 1× PBS (phosphate buffered saline: 8 g L^-1^ NaCl, 0.2 g L^-1^ KCl, 1.44 g L^-1^ Na_2_HPO_4_⋅2H_2_O, 0.24 g L^-1^ KH_2_PO_4_) overnight at 4°C . Samples were washed and concentrated by centrifugation at 1947 g for 1 h. The centrifugation was supported by the addition of Pluronic^®;^ F-127 (0.2 g L^-1^ final concentration), which strongly decreased cells loss. The centrifuged cells were re-suspended in 3 mL 1× PBS and stored at 4°C until further processing.

One year after we performed the fieldwork it has been reported that ^15^N_2_ gas stocks of Sigma are potentially contaminated with NH_3_ ranging from 34 to 1900 μmol NH_3_ per mole N_2_ (Dabundo et al., 2014), which results in the addition of 5–280 nmol L^-1^ ^15^NH_4_^+^ to our incubations at Lago di Cadagno. The specific lot that we used during our experiments was not tested, and it is thus not known whether this lot was also contaminated, and, if yes, to what degree. In order to evaluate whether a potential contamination could have substantially affected our main conclusions, we estimated the impact of a potential contamination as follows: we measured the ^15^NH_4_^+^ uptake in the Cadagno chemocline in a separate experiment in August, 2014, which showed an uptake of 0.19 mol N-NH_4_^+^ per mol C-CO_2_ similar to the Redfield ratio. The bulk C-CO_2_ uptake in our incubation without NH_4_^+^ sampled at 12.9 m depth was 1370 nmol L^-1^ h^-1^ resulting in a NH_4_^+^ uptake potential of 260 nmol NH_4_^+^ L^-1^ h^-1^. Thus, we expect that the added ^15^NH_4_^+^ based on the contamination in the range reported by Dabundo et al. (2014) will be consumed within 1 min to 1 h of incubation, which is only a small fraction of the total incubation time (23 h). We therefore conclude that a possible contamination would only have a minor effect on our results. The incubation in presence of NH_4_^+^ sampled at 14.2 m depth showed no detectable ^15^N enrichment of *C. phaeobacteroides* excluding an effect of the contamination on these results.

In addition, we measured bulk N_2_ fixation rates by preparing the pulse medium according to Mohr et al. (2010) as described above. We experimentally added NH_4_^+^ to some incubations. We used ^15^N_2_ gas provided by Cambridge Isotopes Laboratory (lot I-15312), which has been reported to have consistently low NH_3_ contamination between different lots (0.014–0.052 μmol NH_3_ per mole N_2_) and will not affect N_2_ fixation rate measurements in the nmol L^-1^ d^-1^ range in samples with a background of unlabeled ^14^NH_4_^+^ above 1 μmol L^-1^ (Dabundo et al., 2014). ^15^N enrichment in the biomass and particulate nitrogen was measured with an elemental analyzer connected to an isotope ratio mass spectrometer as described previously (Halm et al., 2009). N_2_ fixation rates were calculated as described previously (Mohr et al., 2010).

### Flow Cell Sorting and Filtration

Sorting of the lake samples was carried out using a MoFlo cell sorter (BeckmanCoulter, USA) equipped with a water-cooled argon-ion laser Innova 70°C (Coherent, Santa Clara, CO, USA). 400 mW excitation at 488 nm was used for analyzing the scatter signals, forward scatter (FSC) and side scatter (SSC). The orthogonal SCC was first reflected by a beam-splitter and then recorded after reflection by a 555 nm long-pass dichroic mirror, passage of a 505 nm short-pass dichroic mirror and a band pass filter 488/10 nm. The red auto-fluorescence (FL3) passed a BLP01-664R-25 long-pass filter (Semrock, Rochester, NY, USA) prior to detection. Amplification of signals was carried out on the logarithmic scale. The SSC was used as the trigger. Fluorescence beads (yellow-green fluorescent beads: 2 μm, FluoSpheres 505/515, F-8827, blue fluorescent beads: 1 μm, FluoSpheres 350/440, Molecular Probes, Eugene, OR, USA, and bright blue Fluoresbrite carboxylate microspheres: 0.5 μm, 360/407, Polyscience, Warrington, PA, USA) were used for calibration. Cells were collected in plastic tubes using the most accurate sort mode (Single Cell One, highest purity 99%).

The sorted cell material was finally transferred to confined areas (ca. 0.3 mm^2^) of membrane filters. Prior to filtration filter pieces of 5 mm diameter were stamped from polycarbonate filters (GTTP, Millipore) sputter-coated with gold/palladium. Sorted cells were transferred onto the surface of these filters by a vacuum-sandwich-procedure. The filters were placed between two parafilm layers that had been previously punched using a 0.8 mm (face down, filtrate side) and a 0.6 mm (face up, feed side) needle. The sandwich was placed onto a glass frit connected to a vacuum filtration unit. The parafilm piece on the filtrate side was smaller than the frit diameter, while the parafilm piece on the feed side fully covered the frit. After applying vacuum, the filter was wetted with water and the sorted cells were pipetted as drops into the hole of the upper parafilm layer.

### CARD-FISH and Marking

CARD-FISH was performed on the filters carrying the sorted cells. The filters were coated with 0.1% low melting agarose pre-warmed to 37°C to avoid cell loss during the hybridization procedure. The HRP-labeled oligonucleotide probe Chlp441 (AAATCGGGATATTCTTCCTCCAC; pos. 441–464) was used to target *C. phaeobacteroides* (Tonolla et al., 2003). An unlabeled competitor probe (AAACCGGGATATTCTTCCTCTAC) targeting *C. chlatratiforme* was added (1:150 v/v) to the hybridization buffer to avoid false positives. Permeabilization, hybridization, and tyramide signal amplification were performed as previously described (Musat et al., 2008). Pre-hybridization and hybridization were performed with 30% formamide at 35°C.

The filter pieces were mounted onto glass slides with a mounting solution containing five parts citifluor AF1 (Citifluor Ltd, UK) and one part vectashield (Vectorlabs, UK), and the general DNA stain Hoechst (10 μg mL^-1^). Areas of interest were marked with laser micro-dissection (PALM micro-dissection, Zeiss 200 M equipped with a 355 nm pulsed UV laser and epifluorescence illumination). For each mark we gathered images of total DNA fluorescence (Dapi filter: ex. 387/11; em. 440/40) and CARD-FISH fluorescence of Oregon Green 488-X (Molecular Probes; GFP filter: ex. 485/20; em. 525/30) that we could use to overlay with the subsequent NanoSIMS images.

### Nanometer-Scale Secondary Ion Mass Spectrometry

The marked areas were analyzed with a NanoSIMS 50L (CAMECA, Gennevilliers Cedex-France) at the Laboratory for Biological Geochemistry of the EPFL Lausanne. The areas were pre-sputtered with a Cs^+^ primary ion beam of 4–4.2 pA (D1–D2) to remove surface contamination, to implant Cs^+^ ions into the sample, and to achieve an approximately stable secondary ion emission rate. A primary Cs^+^ ion beam with a beam current between 1 and 1.2 pA (D1–D3) and a beam diameter of around 100 nm was rastered across the cells for analysis with a dwell time of 5 ms per pixel. Secondary ion images for ^19^F^-^, ^12^C^12^C^-^, ^13^C^12^C^-^, ^12^C^14^N^-^, ^12^C^15^N^-^, and ^32^S^-^ were simultaneously recorded from analysis areas of 30 μm × 30 μm to 40 μm × 40 μm with a resolution of 256 × 256 pixels. Five and six planes from each individual area were measured. Mass resolving power was around 10 000 (Cameca definition), enough to resolve all potential mass interferences from the measured secondary beams.

Analysis of NanoSIMS images was performed with the Matlab-based software Look@NanoSIMS (Polerecky et al., 2012). The images were first corrected for a possible drift of the stage during the measurement and then the counts in each pixel were accumulated over the multiple *z*-planes measured through the cell. We used the fluorescence image and the ^12^C^14^N^-^ ion-image to identify cells and to manually mark these cells as regions of interest (ROI’s). We found that many unidentified cells showed higher ^15^N enrichment than *C. phaeobacteroides* (**Figure 1E**). Special care was taken to not relate pixels belonging to these unidentified cells to *C. phaeobacteroides* ROIs. The accumulated counts, c, were averaged over the area of a ROI and the atom fractions for ^15^N-nitrogen, X(^12^C^15^N)_cell_ = c(^12^C^15^N)_cell_/{c(^12^C^14^N)_cell_ + c(^12^C^15^N)_cell_} and ^13^C-carbon, X(^13^C)_cell_ = c(^13^C^12^C)_cell_/{c(^12^C^12^C)_cell_ + c(^13^C^12^C)_cell_} were calculated for each ROI. These fractions are a measure of the N_2_ fixation and CO_2_ fixation rates, respectively. A standard consisting of cells from a pure culture of *C. phaeobacteroides* grown in the absence of isotopically labeled substrates was prepared and analyzed in the same way. Statistical analysis was performed with Prism 5 Software (GraphPad Software Inc., La Jolla, CA, USA). We used non-parametric statistical tests for within population statistics, because normality tests (D’Agostino-Pearson omnibus K2 and Shapiro-Wilk) indicated that some populations deviated from a Gaussian distribution. Statistical significance between correlation coefficients was tested at vassarstats.net/rdiff.html.

### Isolation of *C. phaeobacteroides*

The N_2_-fixing green sulfur bacterium *C. phaeobacteroides* was isolated from Lake Cadagno in September, 2012. The strain was isolated from water samples collected with a Niskin Bottle from the turbid layer (at around 12 m depth). A water sample of 2 ml was inoculated into 120 ml NH_4_^+^-free, autotrophic growth media for green sulfur bacteria (described below) and grown at 20°C under a Radium BioSun Spectralux®; fluorescent lamp (NL-T8 36W/965/G13, Radium, Germany).

To cultivate the green sulfur bacterium *C. phaeobacteroides*, sulfide-reduced and bicarbonate-buffered media was prepared in a 5 L Widdel-bottle. The basal medium contained per liter of distilled water: 0.5 g KH_2_PO_4_, 0.34 g KCl, 0.5 g MgSO_4_⋅7H_2_O, 0.25 g CaCl_2_. After autoclaving at 121°C for 20 min and cooling to 90°C the headspace was exchanged with 100% N_2_ gas for 15 min. After cooling the medium to room temperature under positive gas pressure, the following anaerobic and sterile solutions were added per liter of basal medium: 30 ml NaHCO_3_ (1 M), 5 ml Na_2_S⋅9H_2_O (0.5 M), 0.5 ml Vitamin B_12_ (1 M) and 1 ml trace element solution for sulfate reducing bacteria. The trace element solution contained 2.1 g FeSO_4_⋅7H_2_O, 13 ml 25% HCl, 5.2 g Na_2_EDTA, 30 mg H_3_BO_3_, 100 mg MnCl_2_⋅4H_2_O, 190 mg CoCl_2_⋅6H_2_O, 24 mg NiCl_2_⋅6H_2_O, 2 mg CuCl_2_⋅2H_2_O, 144 mg ZnSO_4_⋅7H_2_O, and 36 mg Na_2_MoO_4_⋅2H_2_O per liter of distilled water and was adjusted to pH 6.0 with NaOH before autoclaving. Finally, the medium was adjusted to a pH around 6.8 and was stored at room temperature in crimp-sealed serum bottles.

Pure cultures were obtained by repeated application of anaerobic agar plating. Anaerobic plating was realized in 500 ml Schott bottles sealed with black rubber stoppers. Agar (4.5% Agar-Agar, Bacteriology Grade, Applichem) and growth media were mixed 1:3 in an anaerobic bench (240 ml total volume). After setting the agar, 200 μl of diluted culture (about 100 cells) were evenly distributed over the surface. The colonies can be stored dark at 4°C for a few weeks. The identity and purity of the strain was confirmed by CARD-FISH (as described above) and by sequencing a 147 bp fragment of the 16S rRNA with a universal eubacterial primer (27F; 5′-AGAGTTTGATCCTGGCTCAG).

### Stable Isotope Incubations of *C. phaeobacteroides*

Cells were grown in anoxic isolation medium without NH_4_^+^ as described above. The incubation temperature was 20°C and the ambient light intensity was 20 μmol Photons m^-2^ s^-1^ emitted by two fluorescent lamps (Radium BioSun Spectralux®; NL-T8 36W/965/G13). Pre-cultures were initiated from a single colony and used to inoculate experimental cultures in 18 mL medium in a 36 mL serum vial. These cultures were grown for three generations. Subsequently, growing cultures were pulsed with 18 mL medium that was equilibrated with ^15^N_2_ (17 mL L^-1^; Sigma, Lot MBBB0968V) and amended with ^13^C–NaHCO_3_. The final labeling percentage was 50% ^15^N_2_ and 10% ^13^CO_2_. The cultures were incubated for additional 17 h in the presence of the stable isotopes. The incubations were stopped and samples were prepared for NanoSIMS analysis as described above.

The ^15^N_2_ gas lot that we used for these experiments was apparently contaminated with 1900 μmol ^15^NH_4_^+^ per mole N_2_ (Dabundo et al., 2014). We calculated the fraction of ^15^N-NH_4_^+^ molecules fixed into new biomass formed during the incubation per ^15^N-N_2_ molecules fixed using (i) the cell counts before and after the incubation, (ii) particulate nitrogen concentrations measured at the end of the incubation with an elemental analyzer connected to an isotopic ratio mass spectrometer, (iii) the ^15^N_2_ labeling%, (iv) the quantity of N_2_ gas amended to the incubation, (v) the apparent contamination reported by Dabundo et al. (2014), and (vi) by conservatively assuming that all introduced ^15^NH_4_^+^ will be taken up even though its potential starting concentration after label amendment might be at a kinetically limiting concentration of approximately 640 nmol L^-1^. We find that ^15^N-NH_4_^+^ makes up 6 ± 4% (SD, *N* = 3) of the new ^15^N-labeled biomass and thus expect a minor influence on the estimate of phenotypic heterogeneity in N_2_ fixation.

## Results and Discussion

**FIGURE 2:**
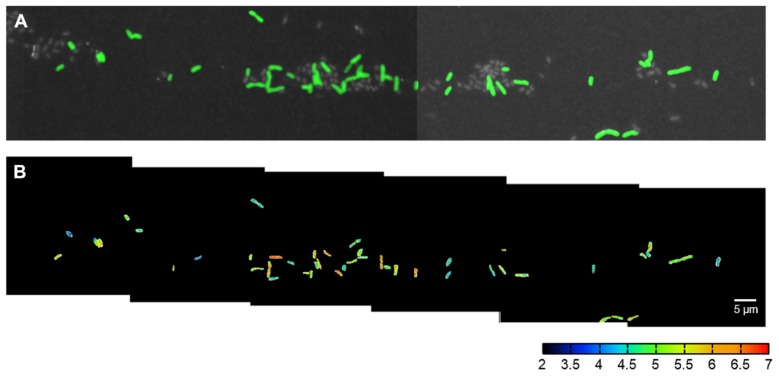
**CARD-FISH identification and ^**15**^N_**2**_ fixation activity of single *C. phaeobacteroides* cells in Lago di Cadagno (turbidity maximum 12.9 m without NH_4_^+^). (A)** Cells that were sorted with flow cell sorting, spotted on a membrane filter, hybridized with CARD-FISH probe targeted against *C. phaeobacteroides* (green), stained with Hoechst general DNA stain (gray), and imaged with epifluorescence microscopy and NanoSIMS. NanoSIMS imaging was done as mosaic-scan overnight. **(B)** The corresponding ^15^N atom fractions [^12^C^15^N/(^12^C^14^N + ^12^C^15^N)]⋅10^-3^ according to the color scale calculated from nitrogen ion counts obtained with NanoSIMS for the cells shown in **(A)**. The cells were segmented on the basis of the CARD-FISH signal in **(A)** and the area outside the cells was set to zero (black) for clarity.

Flow cell sorting of *C. phaeobacteroides* from the Lago di Cadagno microbial community enriched this rare species to abundance levels that allowed the quantification of phenotypic heterogeneity with NanoSIMS (**Figures 1** and **2**). *C. phaeobacteroides* was not visible as a distinct subpopulation based on its cell size and auto-fluorescence because its abundance is very low (1.74 ± 0.14⋅10^3^ cells mL^-1^; SD; *N* = 9; equivalent to 0.2% of total bacteria at 12.9 m) and it shares similar cell properties with other abundant phototrophic bacteria in the chemocline (**Figures 1B,C**). This complicates the localization and optimization of the position of the sorting gate. Thus, we determined the sorting gate based on cell size and auto-fluorescence (ex./em.: 488/>664 nm) of a pure culture isolated from the lake (**Figure 1B**) and applied this gate to lake samples (**Figure 1C**). We detected only very few *C. phaeobacteroides* cells when we investigated sorted populations from adjacent regions (data not shown). This is important, because exclusion of a significant number of cells based on their fluorescence or morphological properties could lead to systematic exclusion of a subpopulation that could potentially be different in its metabolic activity. This issue has to be considered for other enrichment procedures of cells based on flow cell sorting linked to downstream analysis of the sorted population.

Flow cell sorting greatly enhanced our ability to measure many cells within a single NanoSIMS field-of-view (typically 30 μm × 30 μm). If we directly filtered the lake samples onto the membrane filters, the average cell density was only 0.016 cells per 30 μm × 30 μm (field of view). In this case it would have been possible to analyze one cell per field of view if its spatial position was identified and marked before the NanoSIMS measurements. In contrast, flow cell sorting enriched *C. phaeobacteroides* to a relative abundance of 26.5%. Together with identifying and marking spatial positions enriched for cells we were able to measure 7.5 cells per field of view. In total our approach allowed us to measure 252 cells (161 and 91 cells at 12.9 and 14.2 m depth, respectively) in seven individually tuned NanoSIMS images and an overnight mosaic scan (the mosaic scan is shown in **Figure 2**). Therefore our approach allowed quantification of phenotypic heterogeneity for two incubation conditions within 1 day NanoSIMS analysis time.

**FIGURE 3:**
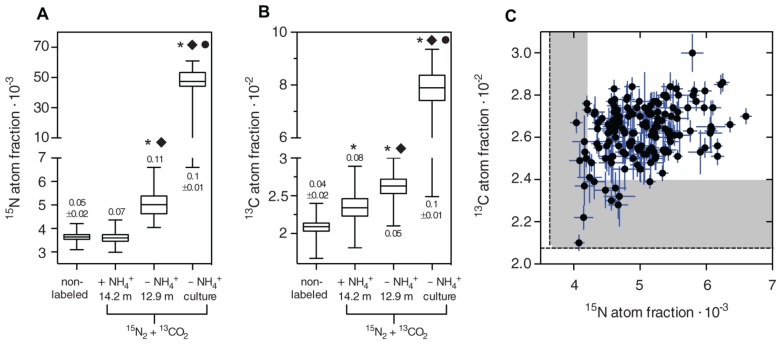
Phenotypic heterogeneity in ^**15**^N_**2**_ and ^**13**^CO_**2**_ fixation of natural and pure culture *C. phaeobacteroides* populations incubated with and without NH_**4**_^**+**^. ^15^N_2_ fixation is represented by the ^15^N atom fraction calculated from the ^12^C^15^N^-^ and ^12^C^14^N^-^ ion counts according to ^12^C^15^N/(^12^C^14^N + ^12^C^15^N). ^13^CO_2_ fixation is represented by the ^13^C atom fraction calculated from the ^13^C^12^C and ^12^C^12^C ion counts according to ^13^C^12^C/(^13^C^12^C + ^12^C^12^C). **(A,B)** Box-and-whisker plots of non-labeled background cells obtained from a *C. phaeobacteroides* pure culture population (five biological replicates, cell number n; *n*_1_ = 49; *n*_2_ = 57; *n*_3_ = 77; *n*_4_ = 34; *n*_5_ = 72) and ^15^N_2_ and ^13^CO_2_ labeled *C. phaeobacteroides* populations from Lago di Cadagno grown in the presence of NH_4_^+^ (14.2 m depth, *n* = 91 cells), from Lago di Cadagno grown in the absence of NH_4_^+^ (12.9 m depth, *n* = 161 cells), and from a *C. phaeobacteroides* pure culture grown in the absence of NH_4_^+^ (three biological replicates, *n*_1_ = 93; *n*_2_ = 100, *n*_3_ = 124). The horizontal line shows the median, the hinges of the box show the 25th and 75th percentile, and the whiskers show the entire range of ^15^N and ^13^C atom fractions in individual cells. The stars indicate a significant difference from the mean of the non-labeled population, the diamond indicates a significant difference from the mean of the Cadagno population incubated with NH_4_^+^, and the circle indicates a significant difference from the Cadagno population incubated without NH_4_^+^ based on a 1-way ANOVA (Kruskal–Wallis test) and Dunn’s multiple comparison test at *p* < 0.05. The numbers above or below the boxes indicate the coefficient of variation ± SD (N according to biological replicates measured) if applicable. **(C)** ^15^N atom fraction plotted against the ^13^C atom fraction for individual cells from the 12.9 m population labeled with ^15^N_2_ and ^13^CO_2_ in the absence of NH_4_^+^ (Spearman *r* = 0.3; *p* < 0.0001). The error bars denote the Poisson counting error of the NanoSIMS measurement indicating the precision of the measurement. The dashed line represents the mean and the shaded area the maximum ^15^N and ^13^C atom fractions of cells in the non-labeled background population.

The NanoSIMS data showed that N_2_ fixation of *C. phaeobacteroides* populations in Lago di Cadagno depended on the availability of NH_4_^+^. Cells incubated in the absence of NH_4_^+^ were significantly enriched in ^15^N_2_, whereas cells incubated in the presence of NH_4_^+^ were not enriched in ^15^N_2_ as compared to a non-labeled control (**Figures 2B and 3A**). Significant levels of single-cell ^13^C enrichment showed that *C. phaeobacteroides* is actively growing regardless of the presence and absence of NH_4_^+^ (**Figure 3B**), confirming that differences in N_2_ fixation activities between both conditions are not due to differences in overall activity. These findings are in line with a previous study that used NanoSIMS to show ^15^N_2_ fixation in *C. phaeobacteroides* incubated with ^15^N_2_ in waters from a depth that does not contain NH_4_^+^ (Halm et al., 2009). In addition, Halm et al. (2009) found *nifH* (coding for a subunit of the N_2_-fixing enzyme nitrogenase) transcripts related to *C. phaeobacteroides* at depth where NH_4_^+^ was present at a concentration as high as 30 μM and also reported significant levels of bulk (total community) N_2_ fixation rates in the presence of NH_4_^+^. This is in contrast to our data showing that *C. phaeobacteroides* did not actively fix N_2_ in the presence of NH_4_^+^. These different results could be due to (i) a change in the physiological properties of *C. phaeobacteroides* between the two sampling events [in 2006 for Halm et al. (2009) and in 2013 for this study], (ii) differences in sampling the depth fine-structure below the chemocline as compared to Halm et al. (2009), (iii) a lower level of N_2_ fixation in the presence of NH_4_^+^ by *C. phaeobacteroides* than in the absence of NH_4_^+^ resulting in N_2_ fixation activities below our detection limit, or (iv) a decoupling between the expression of *nifH* and nitrogenase activity in *C. phaeobacteroides*, as is known in purple non-sulfur bacteria such as *Rhodospirillum rubrum* (Nordlund and Högbom, 2013). In our opinion, the fourth explanation is unlikely because a BLAST search showed that the reported *C. phaeobacteroides* full genome sequences do neither contain annotated proteins involved in post-translational control of nitrogenase [i.e., dinitrogenase reductase ADP-ribosyl transferase (DraT) and dinitrogenase reductase activating glycohydrolase (DraG)] nor gene sequences similar to the gene sequences of those regulatory proteins. Taken together, our data supports previous observations of *C. phaeobacteroides* being capable to fix N_2_ in lakes, but shows that its N_2_ fixation rate is considerably lower or absent in the presence of NH_4_^+^. Moreover, we confirmed the occurrence of bulk ^15^N_2_ fixation below the chemocline (17.3 ± 12.8 nmol N L^-1^ day^-1^; SD; *N* = 5) in the presence of NH_4_^+^ concentrations ranging between 5 and 1000 μM. Consequently, our NanoSIMS data suggests that bacteria other than *C. phaeobacteroides* conduct ^15^N_2_ fixation in the presence of NH_4_^+^ in Lago di Cadagno.

The absolute ^15^N and ^13^C incorporation rates in single *C. phaeobacteroides* cells are low requiring rigorous statistical testing to ensure that differences between cells have a biological origin and are not due to measurement noise. The theoretical precision of the measurement in each cell is represented by the Poisson counting error (σ) that can be obtained from the total accumulated ion counts (μ) in each cell, because the counts during the measurement follow a random variable with a Poisson distribution (Polerecky et al., 2012), in which the variance is equal to the measured mean. The Poisson counting error is calculated for each cell using the relation $\sqrt{1/\mu }.$. This error can serve as a way to visually inspect the measurement noise in relation to the signal (**Figure 3C**), but it cannot be used for standard statistical tests such as ANOVA, because it is not calculated from replicated measurements. We statistically investigated the difference between cells in three steps as suggested by Polerecky et al. (2012): (i) calculation of mean and standard error for each cell by averaging over consecutively measured planes, (ii) 1-way ANOVA and Tukey’s post-test to identify cell pairs that differ significantly from each other in both ^15^N and ^13^C fixation, and (iii) correlation analysis between the accumulated ^15^N and ^13^C atom fractions. The 1-way ANOVA showed that differences between cells were highly significant for ^15^N and ^13^C fixation (both *p* < 0.0001). Tukey’s post-test detected significant differences with a 95% confidence interval in 60 and 12% of all comparisons between cell pairs with respect to ^15^N and ^13^C, respectively. The analysis showed that a difference of 0.425⋅10^-3^ in the ^15^N-atom fraction and 0.255⋅10^-2^ in the ^13^C-atom fraction between two cells is required to statistically resolve a difference. The lower resolution for ^13^C along with the lower number of significant cross-comparisons can be explained by the lower isotopic labeling in ^13^C (2.8%) as compared to ^15^N (20%) during our incubation. Generally, it is advisable to choose the concentration of the isotopic label as high as possible if phenotypic heterogeneity is the focus of the study.

Correlation analysis of accumulated ^15^N and ^13^C atom fractions showed a significant, weak positive correlation between absolute ^15^N and ^13^C fixation for the Lago di Cadagno population (**Figure 3C**; Spearman *r* = 0.3, *p* < 0.0001) and the pure culture (Spearman *r* = 0.42, *p* < 0.0001) incubated in the absence of NH_4_^+^. In contrast, we did not find significant correlations between C and N uptake of single-cells in Lago di Cadagno populations incubated in the presence of NH_4_^+^ (Spearman *r* = -0.08, *p* = 0.46) and unlabeled background populations (Spearman *r* = 0.003 *p* = 0.96). These two correlation coefficients are significantly different from the correlation coefficient of the Lago di Cadagno population incubated without NH_4_^+^ (*p* = 0.0035 and *p* = 0.0044, respectively). This suggests that correlated differences in C and N between cells in the presence of NH_4_^+^ are of biological and not of technical origin. Further, the positive correlation between ^15^N and ^13^C fixation in the absence of NH_4_^+^ suggests that the uptake of both N_2_ and CO_2_ interact and positively facilitate each other in individual cells. However, it remains unclear if cell-to-cell variability is driven by differences in N_2_ fixation activity that translate into differences in CO_2_ fixation or vice versa. One hypothesis would be that inherent stochasticity in the expression of N_2_ fixation genes results in variation in the N_2_ fixation activity between individual cells. According to this hypothesis, these differences would then translate into differences in CO_2_ fixation (i.e., overall growth), because in the absence of NH_4_^+^ growth of an individual cell is limited by its N_2_ fixation activity. An alternative hypothesis would be that cells in the population strongly vary in intracellular components that globally affect gene expression resulting in correlated differences in CO_2_ (i.e., growth) and N_2_ fixation. These differences in intracellular components could be driven by stochastic processes (e.g., unequal distribution of enzymes during cell division) or by genetic differences between cells.

We calculated the coefficients of variation (CV = SD/mean) to compare intra-population variability between populations that have different mean activities. We found that non-labeled pure cultures have a CV of 0.05 ± 0.02 (SD, *N* = 5), which sets the lower bounds for detecting phenotypic heterogeneity as it can be expected that this variability is caused solely by measurement noise. Consequently, the CV of the Lago di Cadagno *C. phaeobacteroides* population incubated in the presence of NH_4_^+^ was in the same range (0.07, *N* = 1), because it showed no significant ^15^N-enrichment. In contrast, the CV of the active Lago di Cadagno *C. phaeobacteroides* population incubated in the absence of NH_4_^+^ (0.11, *N* = 1) was more than twice as high as the CV of the unlabeled population. The CV of a *C. phaeobacteroides* pure culture incubated in the absence of NH_4_^+^ (0.1 ± 0.01, SD, *N* = 3) was in the same range as the actively N_2_-fixing Lago di Cadagno population and significantly different from the non-labeled control (unpaired *t*-test; *p* = 0.0033). We are missing the appropriate number of biological replicates in the samples from Cadagno to show a statistically significant difference between the CV’s of non-labeled controls and the actively N_2_-fixing Lago di Cadagno population. However, the actively N_2_-fixing pure and natural populations have both a rather low heterogeneity in the same range, which indicates that cells of the natural *C. phaeobacteroides* population grow rather homogenously similar to batch culture growth.

It is important to note that we cannot exclude that genetic differences within the natural *C. phaeobacteroides* population are a source for phenotypic heterogeneity observed here. We identified cells based on sequence similarities in their 16S rRNA gene with a species-specific CARD-FISH probe. It is likely that genetic differences are present between cells even if they share a high identity on the 16S rRNA gene as has been shown for wild *Vibrio splendidus* and *Prochlorococcus* populations (Thompson et al., 2005; Kashtan et al., 2014). However, the activity distribution does not show evidence for a distinct functional differentiation in subpopulations, which would be expected if the presence of populations of different genotypes would underlie the phenotypic differences. In the future, it would be desirable to develop tools to integrate single-cell sequencing with phenotypic characterization by NanoSIMS to investigate the effect of genetic diversity on phenotypic heterogeneity in populations of the same bacterial species in an environmental context.

Further, our data show no evidence for a distinct functional differentiation with respect to N_2_ fixation in the absence of NH_4_^+^. The ^15^N data clearly show that the population was not divided into active and inactive subpopulations, but rather spread around an average value. This is in contrast to Halm et al. (2009) who reported that one out of four *C. phaeobacteroides* cells was highly enriched with ^15^N, while the other three measured cells were apparently inactive. A possible explanation for the discrepancy is that Halm et al. (2009) added the ^15^N_2_ tracer as a gas bubble to the incubation, whereas we used a recently developed, modified ^15^N_2_ tracer method in which lake water was pre-equilibrated with ^15^N_2_ and evenly mixed with the sample water (Mohr et al., 2010). The bubble method might lead to strong ^15^N_2_ gradients within the incubation bottle (Großkopf et al., 2012), whereas the modified method mixes ^15^N_2_ evenly at the beginning of the incubation. Consequently, individual cells might be exposed to different ^15^N_2_ concentrations during the incubations by Halm et al. (2009), which artificially established the observed heterogeneity in ^15^N_2_ fixation.

The procedure described in this paper allows the determination of phenotypic heterogeneity in metabolism of a rare species within its natural microbial community. This will pave the way to systematically study phenotypic heterogeneity in metabolism in its natural context and test for the relevance of this phenomenon, which has been extensively and exclusively studied in the laboratory. Our data demonstrate significant differences in N_2_ and CO_2_ fixation between individual cells in a natural population of the green sulfur bacterium *C. phaeobacteroides* and a positive correlation between both activities. Our work also highlights the problems associated with studying phenotypic heterogeneity in nature: measuring a sufficient number of cells, the low activity of cells, and measuring enough replicates to statistically compare the CV’s of populations grown under different environmental conditions. Here, we describe a procedure to measure a sufficient number of cells and provide a solution for the first problem. However, it is important to note that the enrichment of cells in our procedure was based on auto-fluorescence and a pure culture isolate. For many bacteria in nature both might not be available. This limitation might be alleviated by performing CARD-FISH or immunodetection procedures before flow cell sorting (Biegala et al., 2003; Sekar et al., 2004; Mou et al., 2007; Yilmaz et al., 2010; Tada and Grossart, 2014). However, the CARD-FISH protocol involves cell permeabilization and many washing steps that negatively influence cell integrity especially if cells are transferred in solution during or after CARD-FISH to facilitate flow cytometric measurements. Thus, we recommend to sort cells based on morphological characteristics or cell stains followed by CARD-FISH whenever possible. The challenge of low activity can only be overcome by using high isotopic label concentrations or long incubation times. Both solutions might severely impact the physiology of the target organism, because of concentration changes of the metabolic substrate and the bottle effect during long incubation times might lead to changes in the associated microbial community, which potentially interacts with the target population. These factors have to be carefully considered before the experiment and suitable controls should be designed.

## Conflict of Interest Statement

The authors declare that the research was conducted in the absence of any commercial or financial relationships that could be construed as a potential conflict of interest
